# Thyroid Hormone Upregulates Zinc-α_2_-glycoprotein Production in the Liver but Not in Adipose Tissue

**DOI:** 10.1371/journal.pone.0085753

**Published:** 2014-01-21

**Authors:** Rafael Simó, Cristina Hernández, Cristina Sáez-López, Berta Soldevila, Manel Puig-Domingo, David M. Selva

**Affiliations:** 1 Diabetes and Metabolism Research Unit, Institut de Recerca Hospital Universitari Vall d'Hebron, Universitat Autònoma de Barcelona and CIBERDEM (ISCIII), Barcelona, Spain; 2 Service of Endocrinology and Nutrition, Department of Medicine, Germans Trias i Pujol Health Sciences Reseach Institute and Hospital, Universitat Autònoma de Barcelona, Barcelona, Spain; Wayne State University, United States of America

## Abstract

Overproduction of zinc-α_2_-glycoprotein by adipose tissue is crucial in accounting for the lipolysis occurring in cancer cachexia of certain malignant tumors. The main aim of this study was to explore whether thyroid hormone could enhance zinc-α_2_-glycoprotein production in adipose tissue. In addition, the regulation of zinc-α_2_-glycoprotein by thyroid hormone in the liver was investigated. We performed *in vitro* (HepG2 cells and primary human adipocytes) and *in vivo* (C57BL6/mice) experiments addressed to examine the effect of thyroid hormone on zinc-α_2_-glycoprotein production (mRNA and protein levels) in liver and visceral adipose tissue. We also measured the zinc-α_2_-glycoprotein serum levels in a cohort of patients before and after controlling their hyperthyroidism. Our results showed that thyroid hormone up-regulates zinc-α_2_-glycoprotein production in HepG2 cells in a dose-dependent manner. In addition, the zinc-α_2_-glycoprotein proximal promoter contains functional thyroid hormone receptor binding sites that respond to thyroid hormone treatment in luciferase reporter gene assays in HepG2 cells. Furthermore, zinc-α_2_-glycoprotein induced lipolysis in HepG2 in a dose-dependent manner. Our *in vivo* experiments in mice confirmed the up-regulation of zinc-α_2_-glycoprotein induced by thyroid hormone in the liver, thus leading to a significant increase in zinc-α_2_-glycoprotein circulating levels. However, thyroid hormone did not regulate zinc-α_2_-glycoprotein production in either human or mouse adipocytes. Finally, in patients with hyperthyroidism a significant reduction of zinc-α_2_-glycoprotein serum levels was detected after treatment but was unrelated to body weight changes. We conclude that thyroid hormone up-regulates the production of zinc-α_2_-glycoprotein in the liver but not in the adipose tissue. The neutral effect of thyroid hormones on zinc-α_2_-glycoprotein expression in adipose tissue could be the reason why zinc-α_2_-glycoprotein is not related to weight loss in hyperthyroidism.

## Introduction

Zinc-α_2_-glycoprotein (ZAG) is a soluble glycoprotein with a molecular mass of 41 kDa and a crystal structure similar to that of a class I major histocompatibility complex [1]. Its name derives from its tendency to precipitate with zinc combined with its electrophoretic migration to the region of the α2-globulins. ZAG has been found to have a wide range of biological activities but the recent interest in ZAG function comes from its specific lipolytic action and its potential role in body weight regulation [2]–[8]. In fact, ZAG is overproduced by certain malignant tumors and has been characterized as a tumor lipid-mobilizing factor (LMF), which is crucial in the pathogenesis of cancer cachexia [9]–[11].

Apart from an inducer of cachexia in the setting of cancer, ZAG is closely related to obesity. Human adipocytes express and secrete ZAG, with ZAG expression being regulated particularly through TNF-α and the PPARγ nuclear receptor [12]. The action of ZAG is associated with downregulated lipogenic enzymes and upregulated lipolytic enzyme expressions in adipose tissue of mice [13]. Expression of the lipolytic enzymes, such as, adipose triglyceride lipase and hormone-sensitive lipase in white adipose tissue (WAT) were increased two-fold after ZAG administration in rat. In addition, there was almost a two-fold increased expression of uncoupling proteins 1 and 3 in brown adipose tissue and WAT, which could contribute to increase substrate utilization [5]. Serum ZAG levels have been inversely associated with body weight and percentage of body fat in both human subjects and rodents [13]. In addition, we have previously reported that ZAG is downregulated in adipose tissue (both subcutaneous and visceral) and the liver of obese patients [7]. Furthermore, macrophage-associated inflammation may play a significant role in the downregulation of ZAG in adipose tissue in obesity [13]. All these findings point to ZAG not only as a new candidate in the pathogenesis of obesity but also a new therapeutic target [14].

Weight loss is a well-known effect of hyperthyroidism but there is no information whether thyroid hormones could enhance ZAG production in either liver or adipose tissue. In order to shed light to this issue we performed *in vitro* experiments using HepG2 cells and human adipocytes. In addition, in order to have the *in vivo* proof of concept we determined ZAG circulating levels, mRNA and protein levels in both liver and visceral adipose tissue of C57BL/6 mice. Finally ZAG serum levels were measured in a cohort of patients before and after controlling their hyperthyroidism.

## Materials and Methods

### Cell culture experiments

HepG2 hepatoblastoma cells (catalog no. HB-8065; ATCC) were routinely maintained in low-glucose DMEM (catalog no. 11885-084) supplemented with 10% fetal bovine serum and antibiotics (100 U penicillin per milliliter and 100 µg streptomycin per milliliter). For experiments, HepG2 cells were cultured to 80% confluence with low-glucose DMEM and treated over 3 days with vehicle (ethanol) or T_3_ (1 nM, 10 nM or 100 nM). At the end of the experiment, media was collected for analysis and cells were harvested for RNA and protein extraction.

Human preadipocytes were purchased from Tebu-bio (Tebu-bio, Barcelona, Spain) and they were differentiated to mature adipocytes following manufacturer protocol. For experiments, mature adipocytes were cultured in maintenance adipocyte medium and treated daily over the course of 3 days with vehicle (ethanol) or T_3_ (10 nM or 100 nM). At the end of the experiment, media was collected for analysis and cells were harvested for RNA extraction.

### Lipolysis Assay

HepG2 cells were cultured in 96 well plates and maintained in low-glucose DMEM (supplemented with 10% fetal bovine serum and antibiotics) as described above. For lipolysis experiments, glycerol accumulation in the media from HepG2 cells was measured using a Lipolysis Assay Kit (Zen-bio) following the manufacturer instructions. Briefly, HepG2 cells were washed twice with wash buffer and incubated for 6 h with the lipolysis assay buffer (100 µl) containing vehicle, isoproterenol (3 µM) or ZAG (1 µg/ml, 10 µg/ml or 50 µg/ml). After incubation, the 100 µl were plated in a clean 96 well plate and glycerol reagent A (100 µl) was added. The plate was then incubated at room temperature for 15 minutes and optical density of each well was measured at 540 nm.

### Cloning and transient transfections

The cloning of the ZAG proximal promoter region was performed by PCR using the following specific primers: forward XhoI-pZAG (5′-CCCTCGAGTACCTGACCTC AACCTGAGG-3′) and reverse HindIII-pZAG (5′-CCAAGCTTGGTGAATCTACAGGC CAATGG-3′). The PCR product was cloned into the pGL2-luciferase reporter plasmid.

Transient transfections of human *ZAG* promoter-driven luciferase reporter plasmids together with a pCVM-Renilla control plasmid were performed using Lipofectamine 2000 (Invitrogen SA, Barcelona, Spain). Two days after transfection and treatments, luciferase and renilla activity were measured using the Dual-Luciferase Reporter Assay System (Promega, Barcelona, Spain).

### Total RNA preparation and Real-time PCR

After treatments, total RNA was extracted from HepG2 cells, human adipocytes, mouse liver and adipose tissue using TRIzol reagent (Invitrogen SA, Barcelona, Spain). The RNA concentrations were determined by absorbance at 260 nm (A260), and purity was estimated by A260:A280 ratio determination. Reverse transcription was performed at 42 C for 50 min using 3 µg of total RNA and 200 U of Superscript II together with an oligo-dT primer and reagents provided by Invitrogen. An aliquot of the reverse transcription product was amplified in a 25-µl reaction using Power SYBR Green PCR master mix (Invitrogen) with appropriate oligonucleotide primer pairs corresponding to human ZAG (forward primer, 5′-CTTGGCTCACTCAATGACCTC and reverse primer, 5′-CTCCGCTGCTTCTGTTATTC) and human 18S (forward primer, 5′-TAACGAACGAGAC TCTGGCAT and reverse primer, 5′-CGGACATCTAAGGGCATCACAG), mouse ZAG (forward primer, 5′-GAGCCTGTGGGACCTTGGA and reverse primer, 5′-CCTCCCTGGCCCTCTGAA) and mouse 18S (forward primer, 5′-AGGGTTCGATTCCGGAGAGG and reverse primer, 5′- CAACTTTAATATA CGCTATTGG) following a method recommended by Applied Biosystems (Foster City, CA).

### ChIP Assays

HepG2 cells were treated with vehicle or T_3_ (100 nM) for five days. After treatment, we performed ChIP assays with a ChIP-IT kit (Active Motif Inc.) as described previously (15). The antibodies used to immunoprecipite the DNA were the human TRβ1 (sc-114; Santa Cruz Biotechnology Inc.), human TRα/β (C-19; catalog sc-6556; Santa Cruz Biotechnology Inc.). The purified DNA was subjected to PCR amplification (1 cycle of 94°C for 3 minutes, 35 cycles of 94°C for 15 seconds, 59°C for 30 seconds, and 72°C for 45 seconds) using specific primers designed to amplify the region 1 (forward primer 5′-CGAGGTTTCATCATGTTGCCC and reverse primer 5′-CTATTCTAAAGTGA CTGGGGC), region 2 (forward primer 5′-GAAGGCTGGGATTCCACAG and reverse primer 5′-CTCTCAACATGTCCAAGACATG) in the human *ZAG* promoter. As a negative and positive control human *GAPDH* [15] and *PAI-1* [16] promoters were used. The PCR products were resolved by electrophoresis in a 6% acrylamide gel and visualized after ethidium bromide staining.

### Protein extracts

After treatments, protein was extracted from HepG2 using a RIPA buffer supplemented with complete protease inhibitor cocktail (Roche Diagnostics, Barcelona, Spain) at 4 C, followed by centrifugation (12,000 rpm at 4 C) for 10 min to obtain total protein extracts.

### Western blot analysis

Mice blood and HepG2 protein extracts were used for Western blotting with antibodies against human ZAG (1E2; catalog no. sc-21720; Santa Cruz Biotechnology Inc., Santa Cruz, CA) and human PPIA (SA-296; BIOMOL Inc., Plymouth Meeting, PA). Specific antibody-antigen complexes were identified using a horseradish peroxidase-labeled rabbit antimouse IgG or goat antirabbit IgG and chemiluminescent substrates (Pierce Biotechnology Inc., Rockford, IL) by exposure to x-ray film.

### ELISA measurements

ZAG was determined by ELISA (BioVendor, Heidelberg, Germany). The lower limit of detection was 0.673 µg/ml. The intra- and interassay coefficients of variation were 4.7 and 6.6%, respectively.

### Animals

C57BL/6 mice were maintained under standard conditions with food (Global Diet 2018, Harlan Interfauna Iberica, Barcelona, Spain) and water provided *ad libitum* and a 12 h light/dark cycle. For the experiment, male mice (n = 3) were treated with water containing vehicle (ethanol) or water containing T_3_ (0.5 mg/l) for 3 days. Blood samples were taken by saphenous vein immediately before treatment and at the end of treatment when liver and adipose tissue were also collected for RNA extraction. Animals were weighed before and during treatment and no change in weight increase were observed between the two groups. Experimental procedures were approved by the Institutional Animal Use Subcommittees of Hospital Vall d'Hebron Research Institute and the Universitat Autonòma Barcelona.

### Subjects

The study enrolled 27 patients with hyperthyroidism due to Graves' disease (25 women and 2 men). All hyperthyroid patients were initially treated with antithyroid drugs (carbimazole or propylthiouracil) and thyroid function normalized 2–4 months after starting treatment.

Blood samples were obtained after overnight fasting and serum ZAG levels were determined at diagnosis and when thyroid function was normalized. Body mass index (BMI) was calculated as weight in kilograms divided by the square of the height in meters.

Informed written consent was obtained from all participants. Patient's data were obtained and handled according to guidelines of the Human Ethics Committee of our Hospital. The Human Ethics Committee of our Hospital (Comite etic d'Investigacio Clınica, CEIC, Hospital Universitari Vall d'Hebron) waived the need for written informed consent and ethics approval, because information obtained in routine analyses was recorded by the investigator in such a manner that subjects cannot be identified, directly or through identifiers linked to the subjects.

### Statistical analyses

Normal distribution of the variables was evaluated using the Kolmogorov–Smirnov test. Data were expressed as the means ± standard deviation (SD). Comparisons of the continuous variables were performed using paired Student t-test. Spearman correlation coefficient test was used to evaluate correlations. A p value<0.05 was considered significant. Statistical analyses were performed using the SSPS statistical package (SPSS, Chicago, IL, USA).

## Results

### Thyroid hormone (T_3_) increases hepatic but not adipose ZAG production in mice

**Figure 1:**
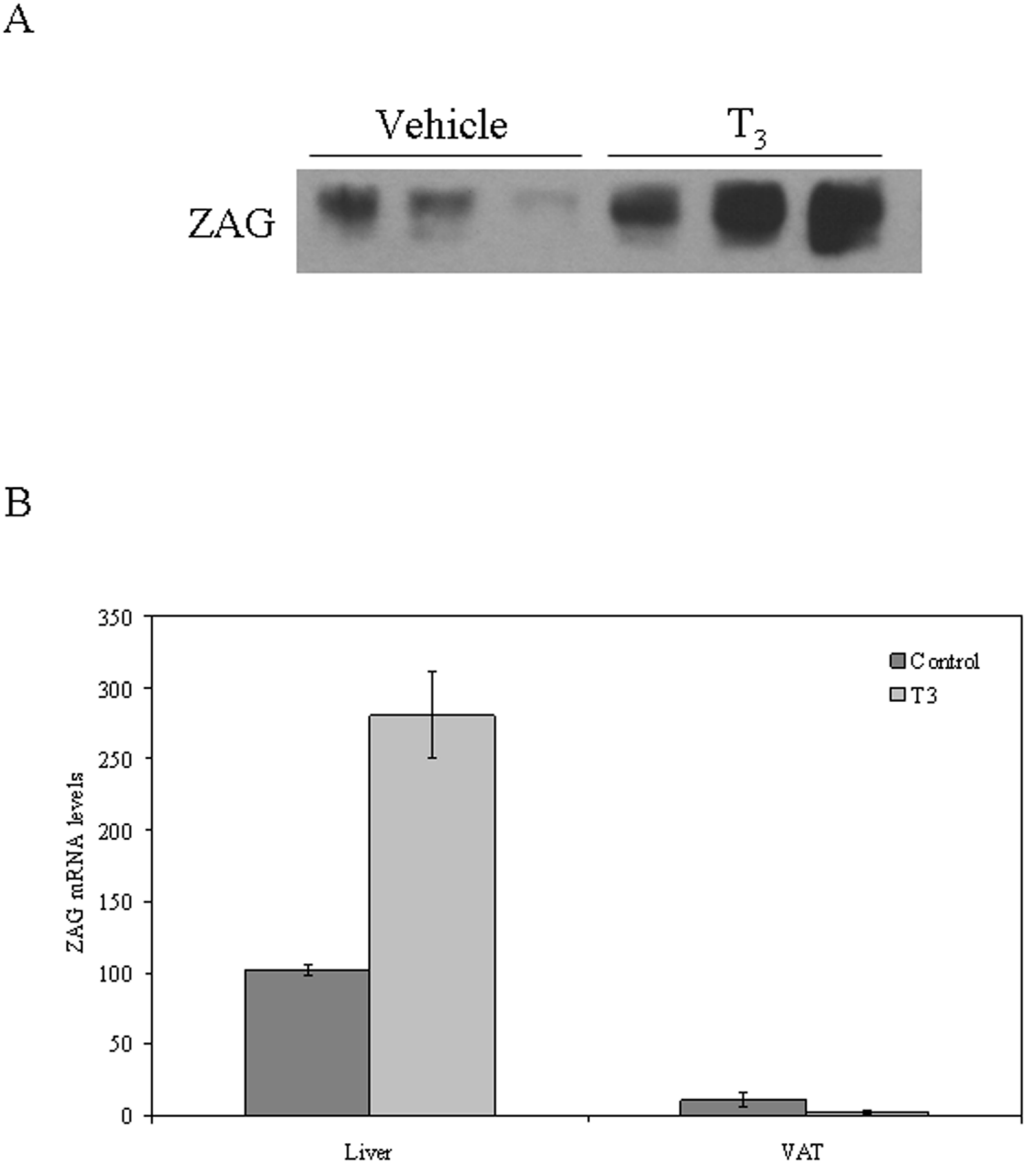
Treatment with T_3_ increases ZAG production by the liver but not in adipose tissue in C57BL/6 mice. (**A**) ZAG blood levels increase in mice treated with T_3_ when compared with vehicle treated mice. (**B**) Analysis of ZAG mRNA expression in liver and adipose tissue of mice treated as in A. Mouse 18S was amplified as an internal control, and values are expressed as percentage relative to the untreated cells. Data are expressed as mean ± SD of triplicates.

In order to explore whether T_3_ increases ZAG production *in vivo* we treated mice (n = 3) daily with T_3_ (0.5 mg/l) or vehicle (ethanol) over the course of 3 days. At the end of the experiment, mice treated daily with T_3_ had increased serum ZAG levels when compared with vehicle treated mice assessed by western blot (Figure 1A).

*ZAG* gene expression (mRNA levels) increased significantly -by 2.5 folds- after acute T3 treatment in the liver but not in visceral adipose tissue (Figure 1B).

### Thyroid hormone (T_3_) increases ZAG production in HepG2 cells but not in human mature adipocytes

**Figure 2:**
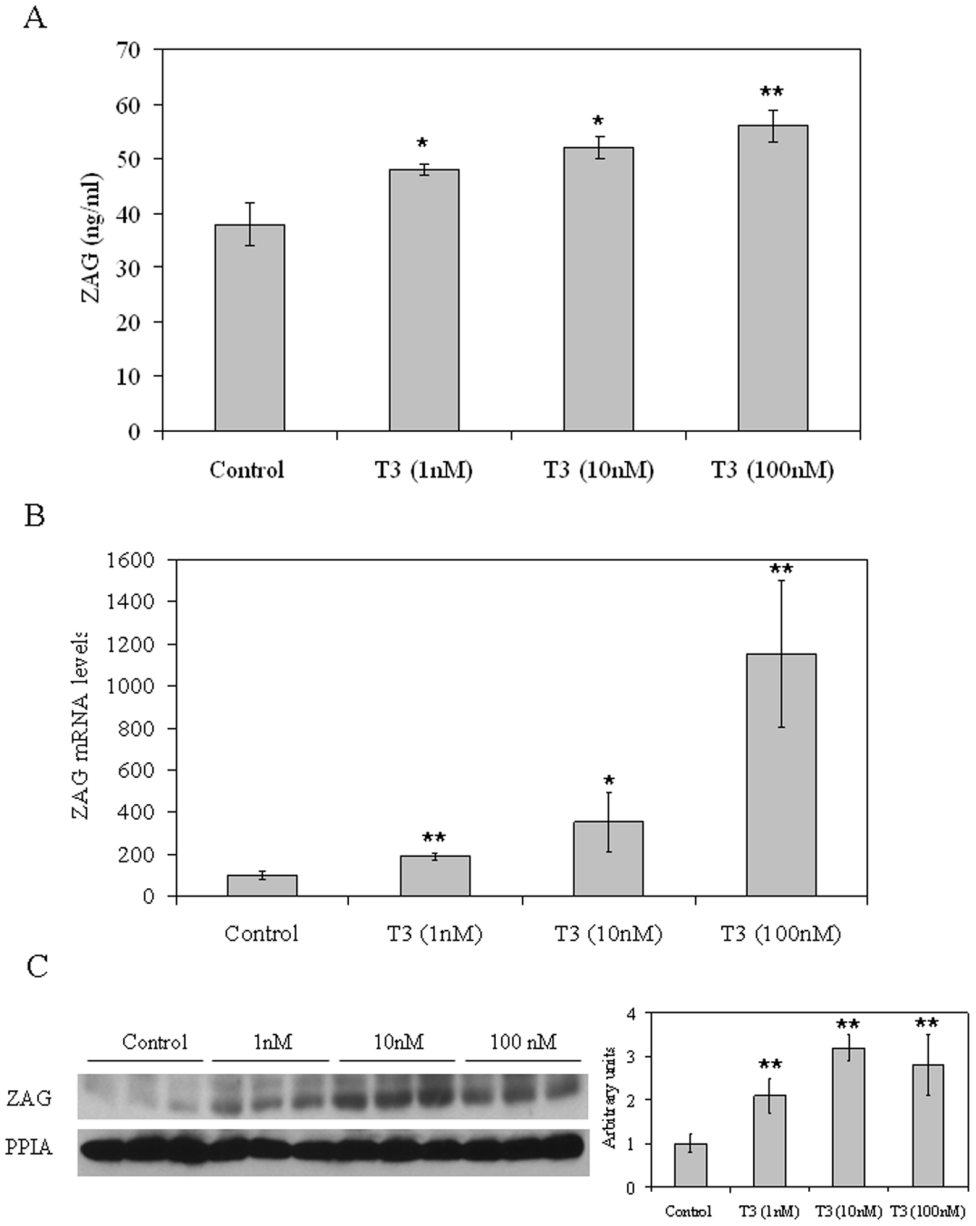
Treatment with T_3_ increases ZAG production in HepG2 cells. (**A**) ZAG media from HepG2 cells treated over the course of 3 days with vehicle (ethanol) or T_3_ (1, 10 and 100 nM) was measured by ELISA. Data are expressed as mean ± SD of triplicates. (**B**) ZAG mRNA levels from HepG2 cells treated as in A. Human 18S was amplified as an internal control, and values are expressed as percentage relative to the untreated cells. Data are expressed as mean ± SD of triplicates. (**C**) Western blot of ZAG and PPIA from extracts of HepG2 cells treated as in A.

We next wanted to examine the effects of different concentrations of thyroid hormone (T_3_) (1, 10 and 100 nM) on ZAG production in the liver. For this purpose vehicle (ethanol) and T_3_ treated HepG2 cells were cultured over the course of 3 days. At the end of the treatments media and cells were harvested for analysis. Vehicle treated cells accumulated ZAG protein in the media over the course of three days, and T_3_ treatment increased ZAG concentrations in the HepG2 media in a dose dependent manner (Figure 2A). Moreover, ZAG mRNA levels were also increased in a dose dependent manner after T_3_ treatment when compared with vehicle treated cells (Figure 2B). Finally, ZAG protein levels were also increased by T_3_ treatment when compared with vehicle treated HepG2 cells (Figure 2C).

**Figure 3:**
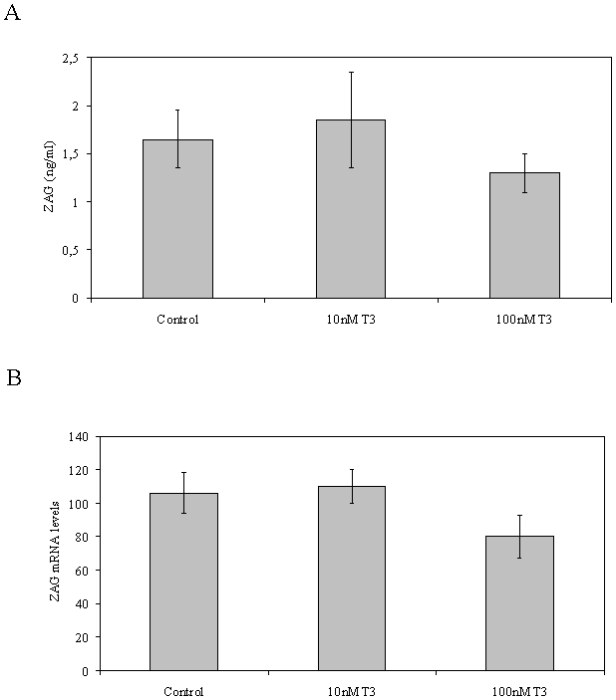
Treatment with T_3_ do not change ZAG production in human adipocytes. (**A**) ZAG media from human adipocytes treated over the course of 3 days with vehicle (ethanol) or T_3_ (10 and 100 nM) was measured by ELISA. Data are expressed as mean ± SD of triplicates. (**B**) ZAG mRNA levels from human adipocytes treated as in A. Human 18S was amplified as an internal control, and values are expressed as percentage relative to the untreated cells. Data are expressed as mean ± SD of triplicates.

We also examined the effects of different concentrations of T_3_ (10 and 100 nM) on ZAG production by human mature adipocytes. Vehicle (ethanol) and T_3_ treated human adipocytes were cultured over the course of 3 days. At the end of the treatments media and cells were harvested for analysis. Vehicle treated adipocytes accumulated ZAG protein in the media but not as much (∼20 fold less) as in HepG2 cultures. In addition, contrary to what occurred in HepG2, T_3_ treatment did not change ZAG accumulation in the media (Figure 3A). Moreover, ZAG mRNA levels were also not changed by T_3_ treatment when compared with vehicle treated cells (Figure 3B).

### Thyroid Hormone (T_3_) increases human ZAG promoter activity in HepG2 cells

**Figure 4:**
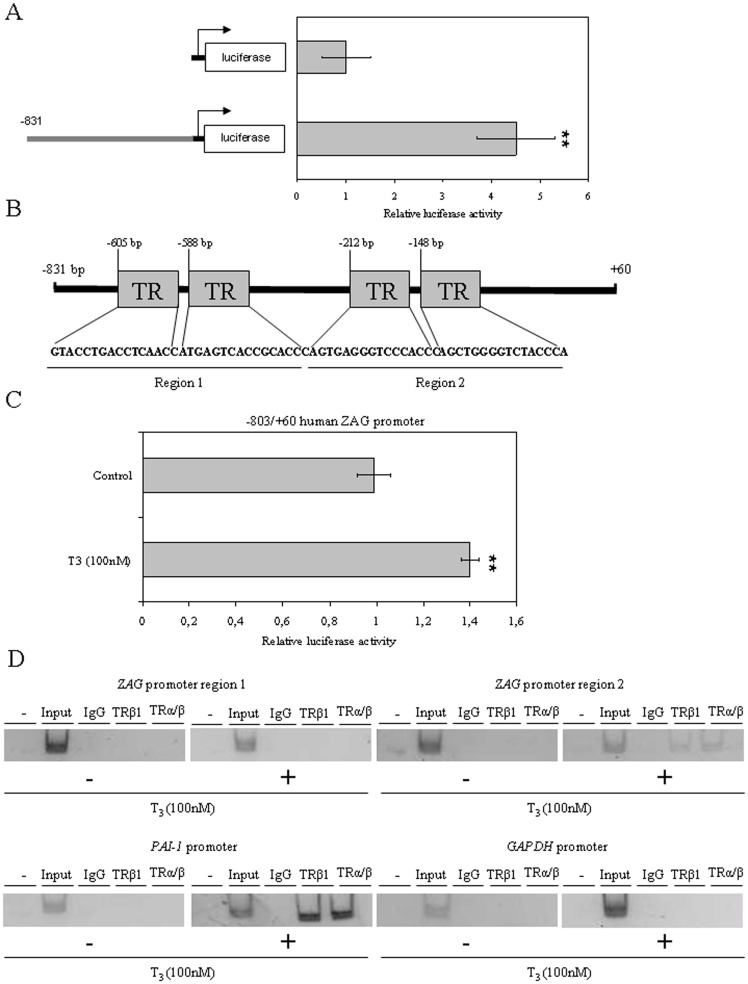
Treatment with T_3_ increases *ZAG* promoter activity in HepG2 cells. (**A**) *ZAG* promoter activity was analyzed in HepG2 cells in luciferase reporter gene assays. Data are expressed as mean ± SD of triplicates. (**B**) Scheme of the human *ZAG* promoter with 4 putative TR binding sites. (**C**) *ZAG* promoter activity was analyzed in HepG2 cells treated with vehicle or T_3_ (100 nM) in luciferase reporter gene assays. Data are expressed as mean ± SD of triplicates. (**D**) ChIP assays of TR binding to the human *ZAG* promoter in HepG2 cells treated with vehicle or T_3_ (100 nM) for 5 days. The *GAPDH* and *PAI-1* promoters were used as a negative and positive control, respectively. Positive PCR controls of sheared genomic DNA templates indicated the integrity of the input DNA used in the ChIP reactions.

We decided to clone the human *ZAG* proximal promoter into the pGL2-luciferase reporter plasmid to analyze its activity in HepG2 cells. The results showed *ZAG* promoter region had 5 times more luciferase activity than the empty pGL2 vector (Figure 4A). Since our results showed that T_3_ treatment increased ZAG production we analyzed the *ZAG* promoter sequence looking for thyroid receptor (TR) binding sites (http://tfbind.hgc.jp/). Indeed, we found 4 TR binding sites in the *ZAG* proximal promoter sequence (Figure 4B). We next analyzed whether T_3_ treatment was able to modify *ZAG* promoter activity in HepG2 cells. The results showed that T_3_ treatment increases *ZAG* promoter activity when compared with vehicle treated cells in luciferase reporter gene assays (Figure 4C).

Finally, to demonstrate the functionality of the TRE present in the human *ZAG* we performed chromatin immunoprecipitation (ChIP) assays using DNA/protein complexes extracted from HepG2 cells treated with vehicle or T_3_ (100 nM) for 5 days. The results indicate that T_3_ treatment promotes the binding of TR to the region 2 of the *ZAG* promoter (Figure 4D).

### ZAG induces lipolysis in a dose-dependent manner in HepG2 cells

**Figure 5:**
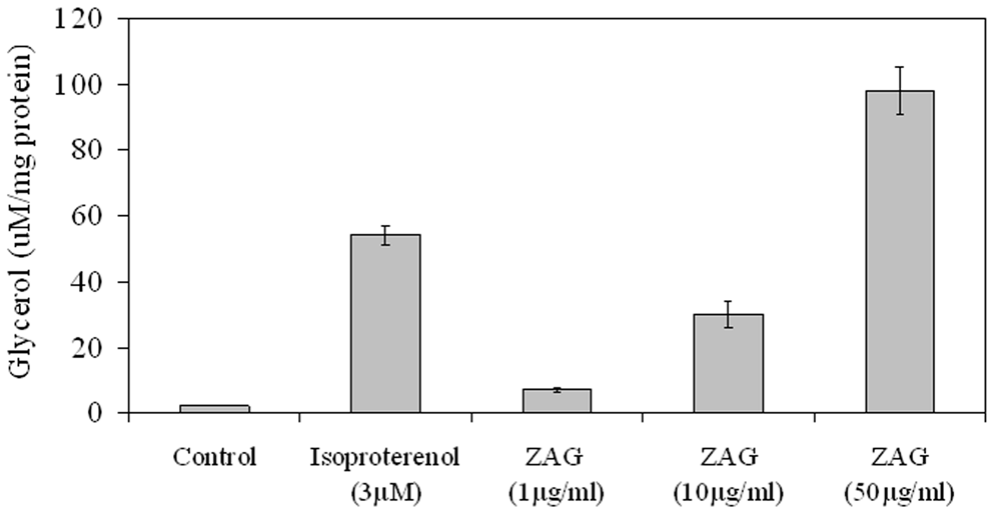
ZAG induces lipolysis in HepG2 cells. Measurement of glycerol accumulation in the medium after treatment for 6(3 µM) and ZAG (1, 10 and 50 µg/ml). Data are expressed as mean ± SD of triplicates.

The lipolytic effect of ZAG on mature adipocytes has been previously described [10]. We wanted to determine if ZAG at the concentrations found in hyperthyroidism patients could induce lipolysis in HepG2 cells. Our results showed that ZAG produces lipolysis in a dose-dependent manner in HepG2 cells (Figure 5).

### Hyperthyroidism increase ZAG serum levels in humans but without being related to weight changes

The clinical and biochemical characteristics of the patients included in this study are shown in table 1. Higher serum levels of ZAG were detected when patients were in hyperthyroidism than when they were with normal thyroid function (47.08±14.04 vs. 32.35±11.35; p<0.0001). A reduction of ZAG serum levels after treatment was detected in all cases. However, we did not find any relationship between circulating ZAG or its decrements after treatment with weigh change.

**Table 1:** Clinical and laboratory features of patients included in the study at diagnosis of hyperthyroidism and at the moment of normalization of thyroid function.

	Hyperthyroidism	Euthyroidism	p
Age (years)	46.42±13.97	-	-
Body Weight (kg)	62.28±7.63	67.83±8.78	0.001
BMI (Kg/m^2^)	24.68±3.57	26.86±3.66	0.001
Free T3 (pg/mL)	3.37±1.23	1.15±0.29	<0.0001
Free T4 (ng/dL)	3.48±1.31	1.41±0.30	<0.0001
TSH (mU/L)	0.008±0.003	2.20±2.30	<0.0001
ZAG (µg/mL)	47.08±14.04	32.35±11.35	<0.0001

## Discussion

The mechanisms regulating ZAG expression are far from being elucidated. It has been reported that PPARγ nuclear receptor activation, glucocorticoids and β3-adrenoreceptor agonists [17]–[19] up-regulate ZAG expression, whereas TNF-α [12], [13], [20]and eicosapentaenoic acid (EPA) [21] have an inhibitory effect in adipose tissue. In addition, we previously reported that TNF-α and IL-6 significantly decrease ZAG production in HepG2 cell cultures [7]. In the present study we provide first evidence that T_3_ upregulates ZAG expression in HepG2 cells. Our experiments showed that thyroid hormone increases hepatic ZAG production in a dose dependent manner. In addition, the cloning of the *ZAG* proximal promoter revealed the presence of four thyroid hormone receptor binding sites and we showed in luciferase reporter gene assays that *ZAG* promoter respond to thyroid hormone treatment. Moreover, we found in ChIP assays that two of the four TRE present in the human ZAG promoter were actually functional. Furthermore, our *in vivo* experiments in mice confirmed the upregulation of ZAG induced by T_3_ in the liver. By contrast, T_3_ did not regulate ZAG production either in primary human adipocyte cultures or in mouse adipocytes. Overall, these findings suggest a differential regulation of ZAG by T_3_ in liver and adipose tissue. This different tissue regulation of gene expression by thyroid hormone has been previously observed in other genes such as pigment epithelium-derived factor (PEDF) [22]. In this regard, one could postulate it is could be possible the existence of an alternative promoter driving ZAG expression in adipose tissue, which might explain the lack of regulation by T_3_. In addition, it is worth recalling that although thyroid hormone receptors (TR) are expressed in both the liver and adipose tissue [23], it is possible that TR activation could differ due to the presence or absence of different co-activators or co-repressors [24]. Moreover, the possibility that the presence of different deiodinases in these tissues also modulates thyroid hormone action should be taken into account [25].

In the clinical setting we observed that the mean reduction of ZAG serum levels after successful treatment of hyperthyroidism was 15 µg/ml (mean decrement ∼30%). Notably, this reduction was observed in all patients after treatment of hyperthyroidism. These results suggest that T_3_ exerts a subtle but consistent modulation of ZAG expression which is sufficient to significantly change its circulating levels. Since T_3_ has no effect on ZAG production in mature adipocytes, the T_3_ induced ZAG upregulation in the liver seems to be the main factor accounting for the increase of ZAG serum levels observed in patients with hyperthyroidism. This finding confirms the idea that, apart from adipose tissue, the liver is an important contributor to systemic levels of ZAG [7].

The lack of relationship between circulating ZAG and weight changes deserves a specific comment. We did not find any relationship between the reduction of ZAG serum levels and the increase of either body weight or BMI after treatment of hyperthyroidism. This finding suggests that systemic levels of ZAG are not a significant factor involved in the weight changes induced by thyroid hormones. In fact, a recent study performed by Mracek et al [26] showed that ZAG levels were not different between cachectic and weight stable cancer patients. In this regard, it should be noted the overexpression of ZAG in the adipose tissue rather than its serum levels is the main determinant of the lipolytic action of ZAG in the cancer-induced cachexia and also in end-stage-renal disease [17], [21], [27]. Since we have found that T_3_ was unable to increase ZAG production by adipose tissue it seems reasonable to deduce that ZAG is not involved in weight loss associated with hyperthyroidism. Taken together, these findings suggest that the autocrine/paracrine action of ZAG in adipose tissue is more important than endocrine action through its systemic levels.

It has recently been reported that iodothyronines induce a reduction of the excess of fat in primary cultures of rat hepatocytes [28]. In addition, an inverse association between serum free thyroxine and hepatic steatosis has been found in a large population based study [29]. In the present study we provide first evidence that ZAG exerts a lipolytic effect in the liver, and this effect was observed in a dose-dependent manner. Notably, the hepatic lipolytic effects were observed in mice using ZAG concentrations detected in hyperthyroid mice. Since T_3_ stimulates ZAG production in the liver it is possible that this is one of the mechanisms involved in fat storage regulation by thyroid hormones in the liver. In this regard, experimental and clinical studies specifically addressed to examining the effect of hyperthyroidism on fatty liver content are needed.

The present study has some limitations. First, the differential effect of T_3_ on ZAG expression in adipose tissue and liver was not confirmed in humans. This was because adipose tissue and liver biopsies were not available. However, it is hard to imagine that this type of biopsy in patients with hyperthyroidism would be approved by any ethical committee. In addition, it should be noted that we have observed the effects of T_3_ on ZAG expression by using levels detected in those patients with hyperthyroidism and we have confirmed in mice the differential effect of T_3_ on ZAG expression. Second, a separate analysis of mature adipocytes and stroma was not performed, and consequently, we cannot rule out that the presence *in vivo* of this stroma-vascular fraction could led to a different T_3_ mediated response of ZAG production by adipose tissue. However, the same effect was observed in adipocyte cultures and in adipose tissue of T_3_ treated mice, thus strongly arguing against this possibility. Third, body fat distribution was not measured. Therefore, specific studies evaluating fat distribution are needed before ruling out any potential role of ZAG in changes of body composition induced by hyperthyroidism.

In conclusion, a differential effect of thyroid hormone on ZAG production was found in hepatic and adipose tissue. T_3_ induced ZAG production by the liver exerts a local lipolytic action and can explain the elevated levels of ZAG induced by hyperthyroidism. By contrast the neutral effect of T_3_ on ZAG expression in adipose tissue is the main factor accounting for the lack of relationship between serum levels of T_3_ and ZAG and suggests that this pathway is not involved in weight loss related to hyperthyroidism. Further studies addressed to examining the effect of T_3_ mediated ZAG overexpression on liver fat storage and body fat distribution seem warranted.
